# Prominent exostosis projecting from the occipital squama more substantial and prevalent in young adult than older age groups

**DOI:** 10.1038/s41598-018-21625-1

**Published:** 2018-02-20

**Authors:** David Shahar, Mark G. L. Sayers

**Affiliations:** School of Health and Sport Sciences, University of the Sunshine Coast, Maroochydore DC, Queensland, 4558 Australia

**Keywords:** Musculoskeletal system, Health care, Health occupations

## Abstract

Recently we reported the development of prominent exostosis young adults’ skulls (41%; 10–31 mm) emanating from the external occipital protuberance (EOP). These findings contrast existing reports that large enthesophytes are not seen in young adults. Here we show that a combination sex, the degree of forward head protraction (FHP) and age predicted the presence of enlarged EOP (EEOP) (n = 1200, age 18–86). While being a male and increased FHP had a positive effect on prominent exostosis, paradoxically, increase in age was linked to a decrease in enthesophyte size. Our latter findings provide a conundrum, as the frequency and severity of degenerative skeletal features in humans are associated typically with aging. Our findings and the literature provide evidence that mechanical load plays a vital role in the development and maintenance of the enthesis (insertion) and we suggest possible associations between aberrant loading of the EOP enthesis, sustained poor posture and EEOP formation. Accordingly, the higher numbers of individuals with EEOP in the 18-30 age group out of all cases examined raises concern about the future musculoskeletal health of this population and suggests a potential avenue for prevention intervention through posture improvement education.

## Introduction

Entheses are the sites of ligament, tendon or joint capsule attachment to a bone^1–3^. A key function of the enthesis is to distribute force over a large area of the bone surface^1,4,5^. Entheses sites are inherently vulnerable to injury as the entheses encompass transitional zones, transferring force between soft and hard tissues^6^. Thus, enthesophyte development may be an adaptive mechanism to further increase the surface area at the tendon/bone interface at sites enduring frequent tensile stress, with bone growth progression taking place in the direction of tensile stress acting on the bone at the point of insertion^2,4^. Enthesophytes often materialize as jagged projections emanating from the bone cortex into the ligament/tendon at the entheses^4,7^. While enthesophytes are often asymptomatic and are not necessarily an indicator for disease in otherwise healthy individuals^4,8^, symptoms at the site of muscular insertion at the EOP have been documented^3,9^. Importantly, enthesophyte formation has been linked to genetic, inflammatory, immunological and biomechanical factors^1,2,5,8^; however these factors do not have an equal influence on entheseal development and the progression of related disorders throughout life in ageing adults^2,10^. Furthermore, enthesophyte formation and enthesitis may be observed on both the axial and appendicular skeleton^3,5^, including the site of muscular attachment on the external occipital protuberance (EOP)^3,11^.

Robust and pronounced cranial features such as cranial thickness, supraorbital torus, a sagittal keel and occipital torus are the hallmarks of early hominin skulls, characteristics that are discussed extensively in the anthropological literature in association with early hominin evolution^12^. With evolutionary changes, such as perfection of bipedalism, cranial balance and equilibrium, and a reduction in the need for powerful mastication has resulted in reduced stress exerted on the skull by muscle tendons and ligaments. Accordingly, the osteological reaction to these external forces has also diminished which has led to the expression of softer cranial attributes in later hominid species^13^. To that end, a significant historical perspective concerning exostosis emanating from the occipital squama at the EOP was provided by the eminent French surgeon and anthropologist Paul Broca (1875)^14^. Broca’s compelling historical documents teach us that despite the typical increased mid sagittal thickness of the cortex at the EOP, the morphology of the periosteum of the occipital squama is predominantly smooth and the EOP is frequently undetectable^14^. Broca’s frustration with the use of the term EOP is depicted by the following text, translated from French in to English: “*Despite the inconvenience, I never dared rejecting this classical name (EOP)… …but I quickly recognized that it was absolutely necessary to reject it, and in my later publications, I substituted it for “inion” (from the Greek, back of the head), which is nowadays fully accepted in France”*^14^.

**Figure 1:**
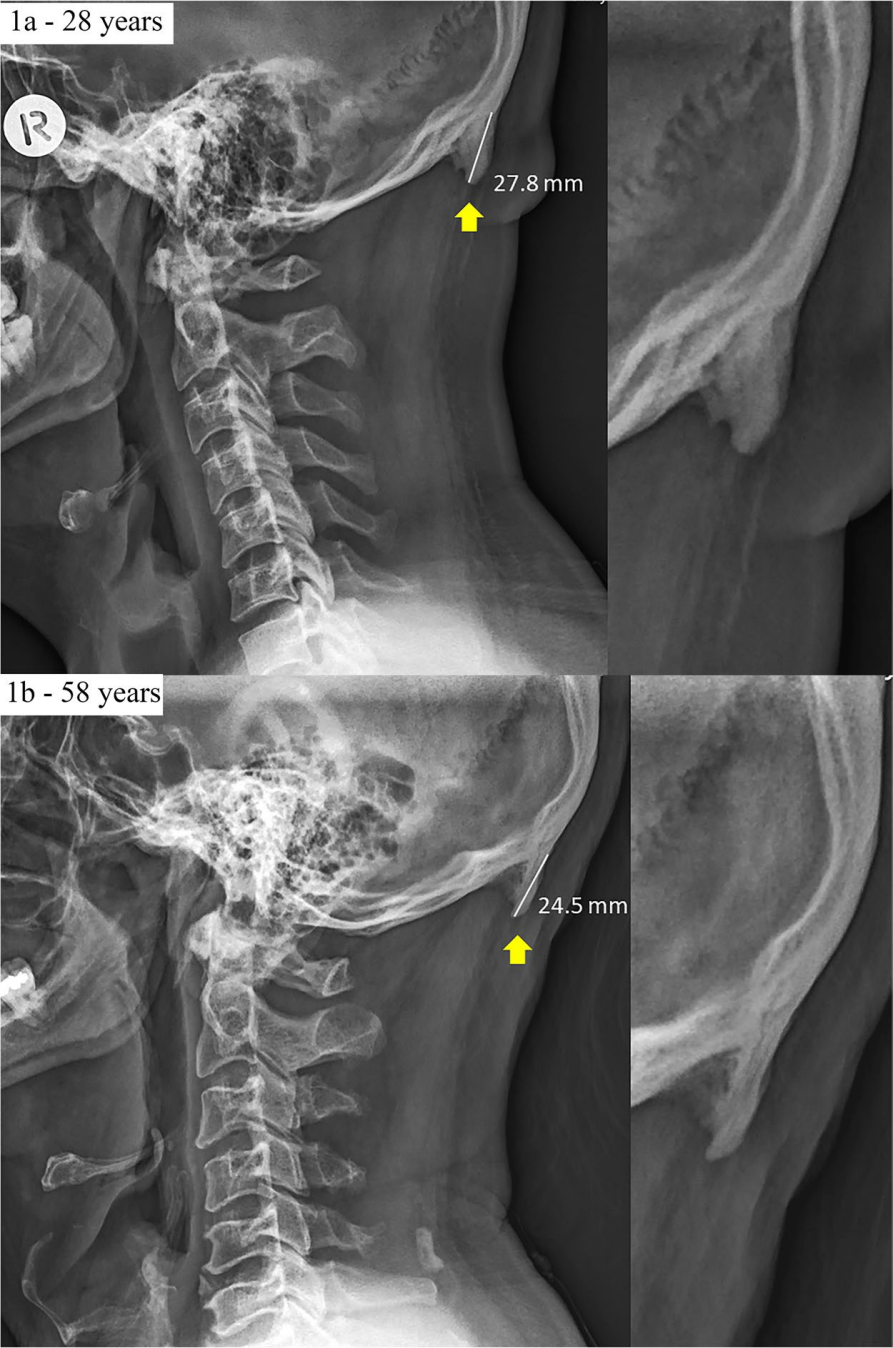
Example radiographs of two male participants (28-years-old and 58-years-old) presenting with large enthesophytes emanating from the occipital squama. These images also include the enthesophyte measurements used throughout this study.

In stark contrast to historical documents and our understanding of the anatomy of the EOP and surrounding structures, we have recently reported on the development of prominent exostosis emanating from this enthesis in over 40% of young adults’ skulls (18–30-year-old, n = 218). To avoid ambiguity, those bony outgrowths were named enlarged EOP (EEOP) only when they have exceeded 10 mm in size (Fig. 1)^15^. In view of the stated paucity of this phenomenon^14,16^, the considerable size of the enthesophytes and their ubiquity with our previous sample was unexpected. More recent scientific literature confirms that enthesophytes are frequently observed on radiographic studies of the ageing asymptomatic population and are part of the normal ageing process^1,8,17^. In contrast, enthesophytes are seen rarely in radiographic findings in young adults, as they are assumed to develop slowly over time^17,18^. Accordingly, to develop our understanding of this phenomenon, the purpose of our current investigation was to determine the distribution of EEOP throughout a wider age group in a larger sample.

## Results

Our current analysis demonstrated the prevalence of EEOP to be 33% of the study population. A binary logistic regression model used to predict the presence of an EEOP was statistically significant (P < 0.001). The model correctly classified the presence of 72.3% of cases (Nagelkerke R2 = 0.26) using the following variables: sex, the degree of forward head protraction (FHP), and age. Odds ratios indicated that being male resulted in 5.48 times increased likelihood of having EEOP (P < 0.001), while every 10 mm increase in FHP resulted in a 1.03 times increased likelihood of having EEOP (P < 0.001).

The mean FHP in the male cases examined was 28 ± 15 mm, while that for the female cases was 24 ± 11mm (P < 0.001). Chi squared analyses (with Adjusted Residuals [AR]) shows that FHP (classified in 10 mm subgroups) was significantly greater in the over 60’s age group than for any of the other age groups (P < 0.001), with FHP >40 mm observed frequently (34.5%) in the over 60s cases (AR=+2.4) (Fig. 3). Additional Chi-squared analyses demonstrated a significant relationship between the distribution of EEOP and age (by decade) (P<0.001). Analysis of these AR data indicate that the presence of EEOP was occurring more frequently than would be expected by chance for both sexes within the 18–30 age-group (males AR= +7.1; females AR= +4.3) (Fig. 4). Conversely, within the other age groups the presence of EEOP for both sexes was distributed as expected (AR> -2.0 and < +2.0) or occurring less frequently than would be expected by chance (AR< -2.0).

**Figure 2:**
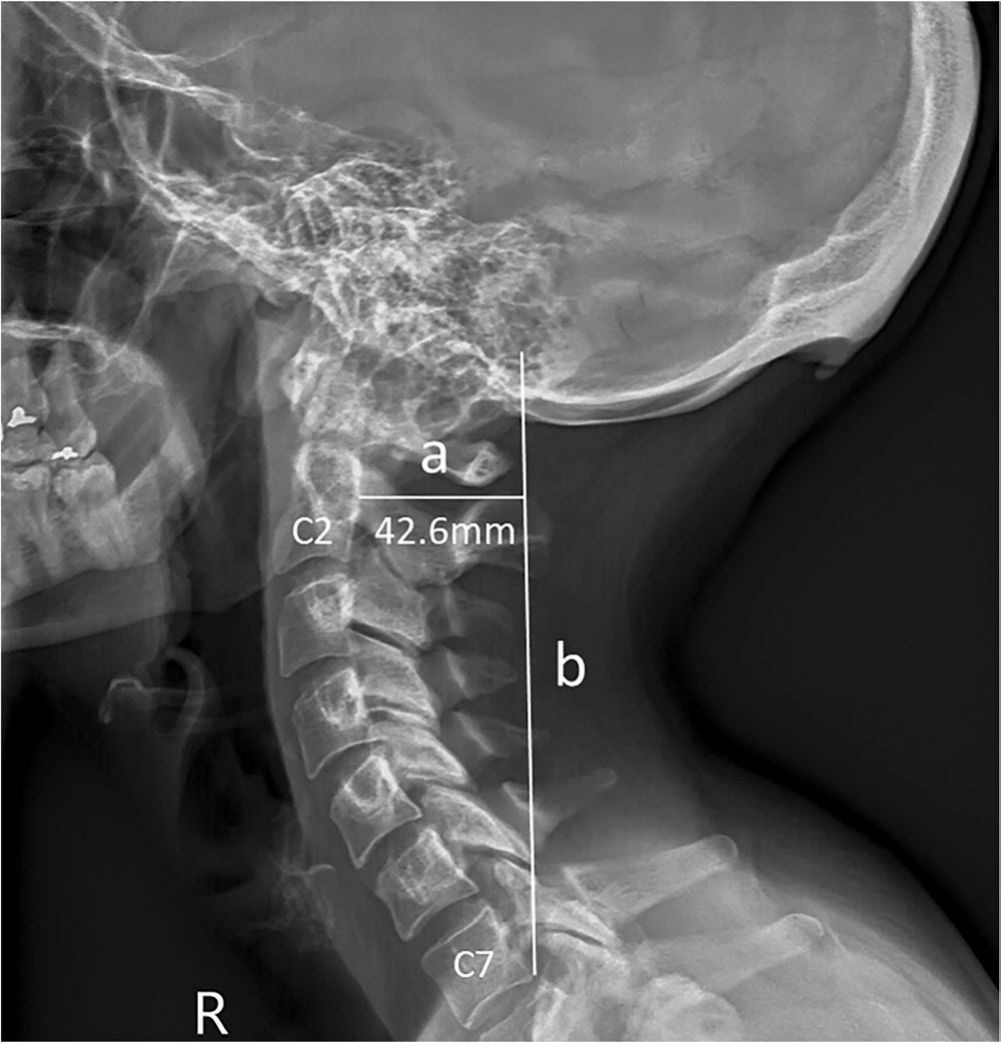
Sample radiographic assessment of forward head protraction (FHP). The extent of FHP was determined by measuring the length (in millimeters) of a horizontal line (a) drawn from the margin of the posterior-superior corner of the body of C2, to a vertical line (b) drawn up from the margin of the posterior-inferior corner of the body of C7 on a lateral cervical radiograph^23^.

**Figure 3:**
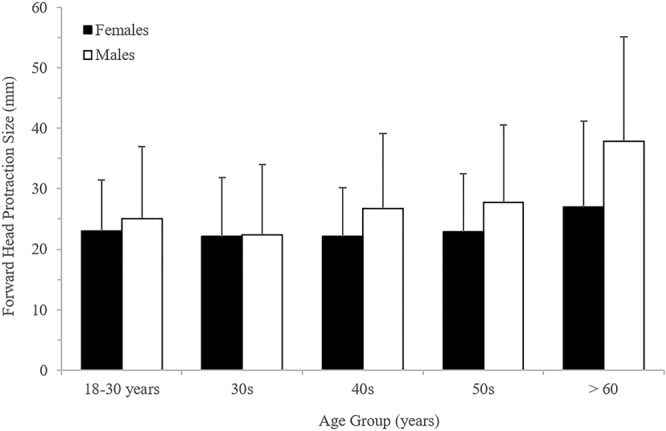
Forward head protraction values across the age groups and sexes.

**Figure 4:**
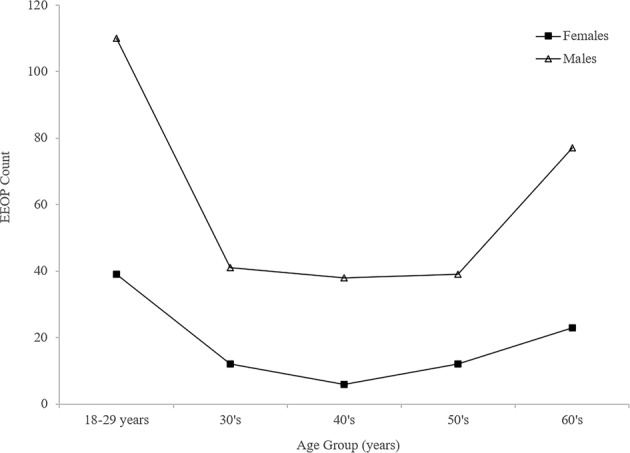
Distribution of the presence of an enlarged external occipital protuberance (EEOP) across age groups within the tested male and female cases.

Unexpectedly, every decade increase in age group resulted in a 1.03 reduction in the likelihood of having EEOP. Chi-squared analyses (*P* < 0.001) demonstrated the 18–30 age-group to be significantly more likely to present with an EEOP, while EEOP was unlikely to occur across any of the other age categories (Fig. 4).

## Discussion

Our findings concerning the influence of both sex and FHP on EOP size were anticipated. The strong association between males and robust cranial traits is acknowledged in the literature^12,14,15,19^, and may be attributed to increased craniocervical mass, muscle power and moment arm lengths^19^. The larger distribution of EEOP in the male population may also be explained by research suggesting that males are more likely to use handheld technology devices for time-consuming gaming and movie viewing, while females are more likely to engage in short duration social activities^20,21^, Furthermore, the synthesis of collagen in response to increased load on tissue was shown to be more moderate in females then males^22^. Not surprisingly, a more pronounced FHP, in both sexes, was correlated with an increase in age groups. Moreover, the mean FHP in our sample was recorded as 26 ±mm, a significantly larger value than the mean recorded in 1996 and prior to the “hand held technological revolution”^23^. Importantly, an increase in FHP increases mechanical load on the posterior craniocervical constituents^24,25^.

Repetitive and sustained mechanical load is required for robust adaptation to take place in tendon properties, as connective tissue adaptive response to load necessitates a slow process of matrix protein (collagen) production^4,6,22^. The development of EEOP may be attributed to, and explained by, the extensive use of screen-based activities by individuals of all ages, including children^25–27^, and the associated poor posture. Musculoskeletal disorders related to poor posture while using computers and tablets have been investigated extensively and were identified as a risk factor for the development of related symptoms at the neck, shoulders and forearms^27–30^. Furthermore, repetitive stress and aberrant posture were reported to be the most common biomechanical risk factors for work-related musculoskeletal disorders of the cervical spine^31^. Importantly, the use of tablet handheld devices was shown to trigger a higher activity level at the upper trapezius and cervical erector spinae^26^. Alarmingly, a survey of university staff and students revealed that participants spend an average of 4.65 hours/day using a hand held mobile device, and that 68% of the participating students reported neck pain^32^. These findings are expected as mechanical load on the cervical musculature was demonstrated to be 3–5 times greater when seated in flexed neck posture than in neutral spine position^25^. A recent systematic review reported that neck-related musculoskeletal conditions amongst mobile and hand-held device users are 17.3–67% more prevalent than any other region of the spine^27^. Obviously, postures that involve sustain forward head flexion or translation will provide similar mechanical stress to those experienced during mobile and hand-held device use. However, many activities involving these postures (e.g. bike riding using drop hand-bars, sleeping supine with a high pillow, etc.) have been prevalent for decades, and therefore cannot provide an explanation to the high prevalence of EEOP in our young adult population. Although the “tablet revolution” is fully and effectively entrenched in our daily activities, we must be reminded that these devices are only a decade old and it may be that related symptomatic disorders are only now emerging^26,27^.

While our findings concerning the influence of both sex and FHP on EOP size were expected, the interaction between age (defined by decade) and EOP size was unforeseen. Our findings contrast directly with reports highlighting the increase in prevalence of degenerative musculoskeletal features in general, and the magnitude of enthesophytes in particular, in aging populations^1,8,17,33^. Our results suggest that the younger age group in our study have experienced postural loads that are atypical throughout the other tested age groups. Similarly, the magnitude of the enthesophytes measured here highlights the substantial mechanical loads acting upon the EOP enthesis. To add perspective to our findings, the Achilles tendon enthesis is subjected to substantial loads due to its role in gait and weight bearing, however, Toumi *et al*.^33^ (n = 1080 males and females, age - 96-year-old) found an absence of large Achilles (dubbed the “premier entheses”) and plantar spurs in the under 40-year old male and female populations. Despite the Achilles tendon entheses being subjected to greater loading than the EOP entheses, it is intriguing that enthesophyte development at the latter appears to be more frequent, more prominent and occurring from early in age.

The greater prevalence of EEOP in our younger population may be explained by research indicating that entheseal development is more reliant on genetic factors during the early days and weeks after birth^10^. More importantly, subsequent development of the enthesis and entheseal transitional zones is determined by mechanical factors, such as repetitive trauma and excessive load acting at the insertion^4,34^. Conversely, enthesophyte formation and inflammation decrease markedly with mechanical load reduction^2^. The aforementioned suggests that excessive forces have been acting on the EOP of our young adult participants and these began during early childhood^15^. Considering our data and the literature on enthesophyte development we hypothesise that a key driver in the development of EEOP is the mechanical load acting on the enthesis due to poor posture and/or poor postural habits. Clearly, our findings should raise concern as morbidity and disability due to musculoskeletal disorders impose increasing physical, social and financial burdens on individuals and societies^35–39^. Accordingly, the mitigation of poor postural habit through prevention intervention may be prudent.

Clearly, the cross-sectional nature of this retroactive case study means that we are unable to draw direct causal links between EEOP formation and other issues such as poor posture and/or the use of mobile phones and other hand-held modern technologies. We acknowledge factors such as genetic predisposition and inflammation influence enthesophyte growth. Similarly, we acknowledge that most of our data were taken retrospectively from a clinician’s database of lateral cervical radiographs, with many individuals therefore originally seeking clinical advice and/or presenting with mild symptomology. Accordingly, despite our exclusion criteria, care should be taken to avoid over generalising these results to an asymptomatic general population. However, the high numbers of EEOP in the 18-30 age group suggests a potential avenue for prevention intervention through posture improvement education in this cohort.

## Methods

This project was provided with full ethics approval from the University of the Sunshine Coast Human Research Ethics Committee. A retrospective analysis of 1200 (18–86-year-old) deidentified lateral cervical radiographic studies was carried out by an experienced observer. In accordance with standard human research ethics procedures informed consent was not required as these data were non-identifiable, with participant’s identity only visible to the clinician. The cohort was divided into age groups according to decades (18–30, 31–40, 41–50, 51–60, >61). Numbers of participants in each age group was: 18–30 n = 300, 31–40 n = 200, 41–50 n = 200, 51–60 n = 200 and >60 n = 300. Participants were chosen from a set time point in the Clinician’s databased (i.e. the most recent) and if they met the selection criteria, with analysis stopping once the required numbers for each age group and gender were reached. Therefore, gender distribution was even amongst all age groups. One half of the 18–30-year-old population was asymptomatic while the rest of the population reported mild musculoskeletal complaints with no specific complaints concerning the EOP. However, it is important to acknowledge that some members of this sample had complaints associated with the cervical spine. The specific symptoms were extracted directly from the patient-intake-form that was completed upon commencing care. Patients that recorded symptomatic complaints greater than mild were excluded from this analysis. The use of radiographs of this mildly symptomatic population is not a limitation, given that the mean EEOP size for the asymptomatic population in our previous assessment (14 ± 7 mm) was significantly greater (*P* = 0.006) than that recorded for the mildly symptomatic population (12 ± 6 mm) in the same study^15^.

All radiographs were obtained by a trained radiographer, at a single chiropractic clinic, by the same digital capturing equipment and with the same capturing techniques. Participants were instructed to stand in their normal posture looking straight ahead, with their right shoulder in contact with the wall mounted ‘Bucky’. The tube-to- Bucky distance was kept constant at 1.5 m. An experienced clinician conducted all radiographic analyses using standard software (Genesis OmniVue® Genesis Digital Imaging, Los Angeles, CA, USA).

During analysis, the clinician could magnify the images for greater accuracy. The size of the EOP was measured using the lateral cervical radiograph and was defined as the distance in millimetres from the most superior point of the EOP (origin) to a point on the EOP that is most distal from the skull (Fig. 1)^15^. These data collection procedures having been shown to be both accurate and reliable (TEM = 1.4 mm, ICC = 0.97)^15^. To avoid any ambiguity, an EOP was classified as enlarged if it exceeded 10 mm and the threshold for recording the size of an EOP was set at 5 mm. Importantly, it is acknowledged that the anatomical level of degeneration is frequently worse than the level of degeneration observed in radiographs^40,41^. Accordingly, any enthesophyte identified using this method is likely to be larger than it appears on the plain radiographs.

Logistic regression analyses were used to ascertain whether the presence of EEOP could be predicted using one or a combination of variables. The differences in EEOP size between the groups and sexes were determined using two-way analysis of variance (ANOVA). Differences between non-parametric variables were determined using Chi-Square analyses, with *AR* testing used to represent the magnitude by which the observed frequency within any cell was above or below the expected value. An *AR* value of ≥2.0 or ≤−2.0 represented a value either substantially more or less (respectively) than the expected value^42^. All statistical analyses were performed using the statistics package SPSS for Windows (version 20), with an alpha level of *P* < 0.05. Data are presented as means (±1 standard deviation [SD]) unless stated otherwise.

## Data Availability

The raw data supporting the findings of this study are available at the USC Research Bank (10.4227/39/5a7104bc0ae51).
