# Author Correction: Prominent exostosis projecting from the occipital squama more substantial and prevalent in young adult than older age groups

**DOI:** 10.1038/s41598-019-49153-6

**Published:** 2019-09-18

**Authors:** David Shahar, Mark G. L. Sayers

**Affiliations:** School of Health and Sport Sciences, University of the Sunshine Coast, Maroochydore DC, Queensland, 4558 Australia

Correction to: *Scientific Reports* 10.1038/s41598-018-21625-1, published online 20 February 2018

This Article contained errors.

In the discussion of the findings, the Article contained language that was speculative of the implications of the study. This was now replaced with the discussion of the limitations of the paper. Therefore, in the Abstract:

“Our findings and the literature provide evidence that mechanical load plays a vital role in the development and maintenance of the enthesis (insertion) and draws a direct link between aberrant loading of the enthesis and related pathologies. We hypothesize EEOP may be linked to sustained aberrant postures associated with the emergence and extensive use of hand-held contemporary technologies, such as smartphones and tablets. Our findings raise a concern about the future musculoskeletal health of the young adult population and reinforce the need for prevention intervention through posture improvement education.”

now reads

“Our findings and the literature provide evidence that mechanical load plays a vital role in the development and maintenance of the enthesis (insertion) and we suggest possible associations between aberrant loading of the EOP enthesis, sustained poor posture and EEOP formation. Accordingly, the higher numbers of individuals with EEOP in the 18-30 age group out of all cases examined raises concern about the future musculoskeletal health of this population and suggests a potential avenue for prevention intervention through posture improvement education.”

And in the Discussion:

“Our findings and the literature provide strong evidence that EEOP in the younger population is a result of increased mechanical load at the enthesis of the EOP, which is probably linked to sustained poor posture. We acknowledge factors such as genetic predisposition and inflammation influence enthesophyte growth. However, we hypothesise that the use of modern technologies and hand-held devices, may be primarily responsible for these postures and subsequent development of adaptive robust cranial features in our sample. An important question is what the future holds for the young adult populations in our study, when development of a degenerative process is evident in such an early stage of their lives?”

now reads

“Clearly, the cross-sectional nature of this retroactive case study means that we are unable to draw direct causal links between EEOP formation and other issues such as poor posture and/or the use of mobile phones and other hand-held modern technologies. We acknowledge factors such as genetic predisposition and inflammation influence enthesophyte growth. Similarly, we acknowledge that most of our data were taken retrospectively from a clinician’s database of lateral cervical radiographs, with many individuals therefore originally seeking clinical advice and/or presenting with mild symptomology. Accordingly, despite our exclusion criteria, care should be taken to avoid over generalising these results to an asymptomatic general population. However, the high numbers of EEOP in the 18-30 age group suggests a potential avenue for prevention intervention through posture improvement education in this cohort.”

Additionally, to aid the readers, additional methodological details were included in the first two paragraphs of the Results:

“Our current analysis demonstrated the prevalence of EEOP to be 33% of the total population. Logistic regression analysis indicated the presence of an EEOP to be significantly predicted (72.3%; P < 0.001) using the following variables: sex, the degree of forward head protraction (FHP), and age. Sex was the primary predictor with males being 5.48 times more likely to have EEOP than females (P < 0.001).

The extent of FHP (Fig. 2) was a significant component in the prediction of EEOP, where an increase in FHP resulted in a 1.03 times increased likelihood of having EEOP (P < 0.001). Our data also reveals that sex and age are significant factors with the mean FHP in the male population being 28 ± 15 mm while that for the female population was 24 ± 11 mm (P < 0.001). Additionally, FHP was significantly greater in the over 60 s age group than for any of the other age groups (P < 0.001), with FHP > 40 mm observed frequently (34.5%) in the over 60 s population (AR = +2.4) (Fig. 3). Accordingly, increases in age groups of both sexes are correlated with a more pronounced FHP.”

now reads:

“Our current analysis demonstrated the prevalence of EEOP to be 33% of the study population. A binary logistic regression model used to predict the presence of an EEOP was statistically significant (P < 0.001). The model correctly classified the presence of 72.3% of cases (Nagelkerke R2 = 0.26) using the following variables: sex, the degree of forward head protraction (FHP), and age. Odds ratios indicated that being male resulted in 5.48 times increased likelihood of having EEOP (P < 0.001), while every 10 mm increase in FHP resulted in a 1.03 times increased likelihood of having EEOP (P < 0.001).

The mean FHP in the male cases examined was 28 ± 15 mm, while that for the female cases was 24 ± 11 mm (P < 0.001). Chi squared analyses (with Adjusted Residuals [AR]) shows that FHP (classified in 10 mm subgroups) was significantly greater in the over 60’s age group than for any of the other age groups (P < 0.001), with FHP > 40 mm observed frequently (34.5%) in the over 60 s cases (AR = +2.4) (Fig. 3). Additional Chi-squared analyses demonstrated a significant relationship between the distribution of EEOP and age (by decade) (P < 0.001). Analysis of these AR data indicate that the presence of EEOP was occurring more frequently than would be expected by chance for both sexes within the 18–30 age-group (males AR = +7.1; females AR = +4.3) (Fig. 4). Conversely, within the other age groups the presence of EEOP for both sexes was distributed as expected (AR > −2.0 and < +2.0) or occurring less frequently than would be expected by chance (AR < −2.0).”

The original Figure 4 showed EEOP counts in percentages, calculated as the number of observed EEOPs within male/female group divided by a total number of observed EEOPs. The original version of Figure 4 is reproduced below as Figure 1.

**Figure 1:**
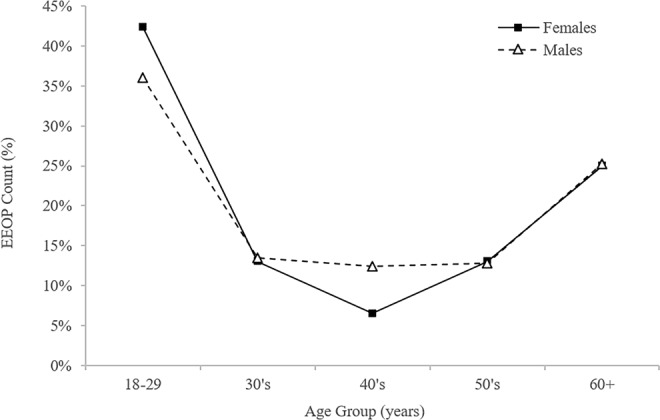
.

Additionally, the figure legend incorrectly stated that these represent prevalence of EEOP in both sexes across the age groups. These were showing the proportion of female/male patients with EEOP among the total EEOP cohort. The figure was replaced with a version that for clarity reports EEOP counts in absolute numbers. The legend was also corrected:

“Prevalence of an enlarged external occipital protuberance (EEOP) in both sexes across the age groups.”

now reads:

“Distribution of the presence of an enlarged external occipital protuberance (EEOP) across age groups within the tested male and female cases.”

The raw data underlying the study were originally deposited, but not signposted in the Article. This is now corrected and the Data Availability section:

“The authors declare that all data supporting the findings of this study are available in the article.”

now reads:

“The raw data supporting the findings of this study are available at the USC Research Bank (10.4227/39/5a7104bc0ae51).”

Finally, the Competing Interest statement was also updated and:

“The authors declare no competing interests.”

now reads:

“David Shahar provides posture related services as a chiropractic clinician and posture related advice and products through drposture.com. Mark Sayers declares no competing interests.”

